# Association of postoperative delayed bowel function recovery with weight and muscle loss at 6 months in colorectal cancer patients

**DOI:** 10.3389/fnut.2026.1827831

**Published:** 2026-08-07

**Authors:** Shuaichao Li, Shaoying Li, Tao Meng, Zhengjie Gao, Hailong Feng

**Affiliations:** Department of General Surgery, The First Affiliated Hospital of Xinxiang Medical University, Weihui, Henan, China

**Keywords:** body weight, colorectal cancer, delayed bowel function recovery, muscle mass, nutritional status

## Abstract

**Background:**

Delayed bowel function recovery (DBFR) is a common postoperative complication after colorectal cancer (CRC) resection. This study aimed to investigate the association between postoperative DBFR and 6-month weight and muscle mass loss in patients with CRC, to inform optimized postoperative prognostic management.

**Methods:**

A total of 618 patients with CRC were prospectively enrolled. Body weight and five anthropometric parameters (including mid-upper arm circumference) were measured within 3 days before surgery. Skeletal muscle cross-sectional area at the L3 vertebral level was quantified using preoperative abdominal CT scans, with the skeletal muscle index (SMI) subsequently calculated. DBFR was defined as the absence of first flatus and defecation within 72 postoperative hours. All participants completed a 6-month follow-up, and body weight, SMI and the five anthropometric measurements were reassessed at the follow-up endpoint. Multivariate linear regression models were applied for statistical analyses.

**Results:**

Postoperative DBFR was nominally or significantly correlated with lower final body weight, reduced SMI, decreased mid-upper arm circumference, calf circumference and hip circumference, as well as reduced triceps skinfold thickness at 6-month follow-up. Notably, DBFR was significantly associated with larger declines from baseline to follow-up in body weight, SMI, mid-upper arm circumference, calf circumference, waist circumference, hip circumference and triceps skinfold thickness.

**Conclusion:**

Postoperative DBFR correlates with medium-term weight and skeletal muscle loss in patients undergoing CRC surgery. Upon further external validation, DBFR may act as a predictive risk factor for these unfavorable nutritional outcomes.

## 1 Introduction

Colorectal cancer (CRC) contributes to over 2 million new cases and more than 900,000 deaths annually worldwide (1, 2). Its incidence has been steadily rising in China and most developed countries, rendering CRC one of the most prevalent malignant tumors globally. As a leading malignancy of the digestive system, CRC severely compromises patients’ life quality and imposes a substantial socioeconomic burden, constituting a major global public health challenge (3).

Approximately 60–80% of patients with CRC present with stage I–III disease without distant metastasis, for whom surgical resection serves as the primary curative treatment (4). Delayed bowel function recovery (DBFR) is a prevalent postoperative complication after CRC surgery, with a reported incidence of 15–35% that varies by surgical approach, patient baseline status, and perioperative management (5, 6). This complication not only prolongs hospital stay and increases medical costs but also predisposes patients to adverse events including intestinal obstruction and surgical site infection, thereby hindering postoperative recovery and impairing long-term quality of life.

Furthermore, postoperative weight loss and muscle mass depletion are common in CRC patients, both of which are well-established prognostic indicators. Accumulating evidence has demonstrated that reduced body weight or low muscle mass is strongly associated with higher risks of tumor recurrence and metastasis, as well as shortened disease-free and overall survival (7–10). Accordingly, accurate identification and timely intervention of postoperative body composition changes are clinically critical for improving CRC patients’ prognosis.

To date, the specific relationships between postoperative DBFR and subsequent weight and muscle mass loss in CRC patients remain poorly defined. Nevertheless, this association is biologically plausible: DBFR may disrupt postoperative nutrient absorption and metabolic homeostasis, thereby exacerbating adverse body composition alterations. If verified, DBFR may serve as a novel clinical marker for stratifying the risk of postoperative weight and muscle loss, facilitating early targeted intervention.

In this study, we enrolled a large cohort of patients undergoing CRC resection, documented postoperative DBFR occurrence, and performed a 6-month follow-up to assess postoperative weight loss and muscle mass reduction. We systematically analyzed the associations between DBFR and these two body composition outcomes. The study flowchart is presented in Supplementary Figure 1. The findings may provide novel evidence for the prognostic value of DBFR and offer insights into optimizing postoperative risk stratification and early intervention strategies for CRC patients.

## 2 Materials and methods

### 2.1 Ethical requirements

This study was approved by the Ethics Committee of The First Affiliated Hospital of Xinxiang Medical University, and all procedures were performed in accordance with the Declaration of Helsinki and relevant ethical guidelines (11).

### 2.2 Patient inclusion

From January 1, 2017, to June 30, 2024, consecutive patients receiving radical CRC resection at the Department of General Surgery, The First Affiliated Hospital of Xinxiang Medical University, Henan, China, were enrolled.

The inclusion criteria were as follows:

(1) Histopathologically confirmed primary colorectal cancer;
(2) Stage I–III TNM stage without distant metastasis who underwent elective curative resection (12, 13);
(3) Absence of psychological, psychiatric or cognitive dysfunction; patients capable of understanding study protocols and finishing all scheduled assessments;
(4) No concurrent severe life-threatening disorders (e.g., acute myocardial infarction, synchronous extracolonic malignancies);
(5) Preexisting chronic illnesses including essential hypertension and type 2 diabetes were stably controlled;
(6) Written informed consent obtained; participants agreed to body weight testing within 3 preoperative days and at 6 postoperative months, and permitted access to inpatient charts and outpatient follow-up data including abdominal CT scans.

The exclusion criteria were as follows:

(1) Unconfirmed CRC diagnosis or ineligible for radical resection;
(2) Coexisting non-CRC conditions that may interfere with outcome measurements or raise participation risks;
(3) Incomplete medical records precluding valid data analysis;
(4) Loss to follow-up, cancer recurrence, participant withdrawal or other causes resulting in missed 6-month postoperative measurements.

### 2.3 Baseline data collection

Admission for surgical treatment was defined as the baseline of this study. Researchers collected the following baseline data by reviewing medical records and conducting face-to-face interviews: (1) Demographic data; (2) Personal history; (3) Disease history; (4) Family history; (5) Preoperative serological indicators; (6) Tumor characteristics and treatment measures.

In terms of personal history indicators, smoking was defined as smoking at least 1 cigarette per day for a duration of ≥12 months; drinking was defined as consuming alcoholic beverages (e.g., liquor, beer, wine) at least 3 times per week for ≥12 months; a low-fiber diet was defined as a daily dietary fiber intake of <25 g in the past year, assessed by a food frequency questionnaire (14); high red meat intake was defined as an average daily intake of red meat (e.g., beef, pork, lamb) of ≥100 g in the past year, evaluated via a food frequency questionnaire (14); a sedentary lifestyle was defined as accumulating <150 min of moderate-to-vigorous physical activity per week and spending ≥8 h per day sitting or lying down in the past year; and staying up late was defined as regularly going to bed after 00:00 (midnight) on ≥3 days per week with a daily sleep duration of <6 h in the past year.

### 2.4 Baseline body weight and muscle mass measurement

Within 3 days before surgery, each patient’s body weight (unit: kg) was measured using a calibrated electronic scale in the morning after an overnight fast.

The latest abdominal CT images at the L3 vertebral level obtained within 3 days before surgery were retrieved for each patient. The mid-vertebral slice of L3 was selected for muscle analysis. Skeletal muscle segmentation was performed using ImageJ 1.53c software (developed by the U. S. National Institutes of Health). A standardized Hounsfield unit (HU) threshold of −29 to +150 HU was used to define skeletal muscle tissue. The region of interest was manually delineated along the anatomical boundary of skeletal muscle, while subcutaneous fat, visceral fat, bone, and bowel tissue were carefully excluded. The skeletal muscle cross-sectional area at the L3 level (L3-SMA) was automatically calculated by the software. The skeletal muscle index (SMI, cm^2^/m^2^) was further calculated using the formula: SMI = L3-SMA / (height × height) (15).

All CT measurements were independently performed by two senior radiologists, who were blinded to patient grouping, clinical characteristics, and follow-up outcomes. The inter-observer reproducibility was assessed using the intraclass correlation coefficient (ICC). An ICC value > 0.90 was defined as excellent agreement, under which the average of the two independent measurements was adopted for subsequent analysis. If the ICC failed to reach 0.90, the two radiologists re-examined the CT images together, repeated the segmentation and measurement, and reached a consensus until a satisfactory agreement was achieved.

Meanwhile, five anthropometric indicators were measured using standardized methods. Specifically, mid-upper arm circumference (cm) was measured with the patient standing naturally and the upper arm relaxed and hanging down: a tape measure was wrapped horizontally around the midpoint of the upper arm, closely adhering to the skin without compressing the tissue. Calf circumference (cm) was measured with the patient standing with feet shoulder-width apart: the tape measure was wrapped horizontally around the thickest part of the calf, ensuring it was perpendicular to the calf’s long axis. Waist circumference (cm) was measured with the patient fasting and standing with feet separated by approximately 30 cm: the tape measure was wrapped horizontally around the abdomen at the umbilical level, with tightness allowing the insertion of 1–2 fingers. Hip circumference (cm) was measured with the patient standing with legs together: the tape measure was wrapped horizontally around the most prominent part of the buttocks, kept level and closely attached to the skin. Triceps skinfold thickness (mm) was measured with the patient’s upper arm naturally hanging down: a skinfold caliper was used to vertically pinch the subcutaneous fat at the posterior midpoint of the line connecting the acromion and olecranon process.

### 2.5 DBFR determination

Postoperatively, attending surgeons and researchers dynamically monitored patients’ abdominal signs, as well as the recovery of intestinal flatus and defecation. Given that the 72-h postoperative time point is a widely accepted threshold for defining gastrointestinal function recovery and that the return of spontaneous flatus and defecation serves as the core objective indicator for assessing bowel function, postoperative DBFR in patients with CRC was defined as the absence of spontaneous flatus and defecation at 72 h after surgery, regardless of abdominal distension, abdominal pain or other abdominal symptoms. This broadly inclusive clinical definition can capture patients with mild, moderate, and severe postoperative abnormalities in gastrointestinal function recovery.

### 2.6 Follow-up

All patients received 6-month follow-up after hospital discharge. Home visits were scheduled at the end of the 2nd and 4th post-discharge months, and outpatient review was arranged at the 6-month endpoint. Disease status and ongoing treatment were recorded at all three follow-up visits. The simplified Food Frequency Questionnaire (FFQ-25) was completed on three occasions to evaluate daily dietary fiber, protein and energy intake; mean values were computed for subsequent analyses. Prior publications have verified acceptable nutrient validity and favorable test–retest reliability for FFQ-25 and comparable abbreviated food frequency questionnaires, supporting its robust methodological validation (16, 17).

At the scheduled 6-month postoperative follow-up, body weight and the five aforementioned anthropometric parameters were reassessed. All patients underwent routine abdominopelvic contrast-enhanced CT per standard CRC surveillance guidelines; these CT scans were used to recalculate L3-SMA and SMI following the previously described protocol. Absolute changes in body weight, L3-SMA, SMI and each anthropometric measure from preoperative baseline to the 6-month follow-up were calculated for statistical analysis.

### 2.7 Statistical analysis

Statistical analyses were conducted as described previously (18, 19).

The Kolmogorov–Smirnov test verified normal distribution for all continuous variables (data not shown). Continuous variables were expressed as mean ± standard deviation (SD), with between-group comparisons via independent-samples t-test. Categorical variables were presented as frequencies (proportions), and intergroup disparities were compared using the χ^2^ test. Statistical significance was defined at *P* < 0.05.

**Figure 1:**
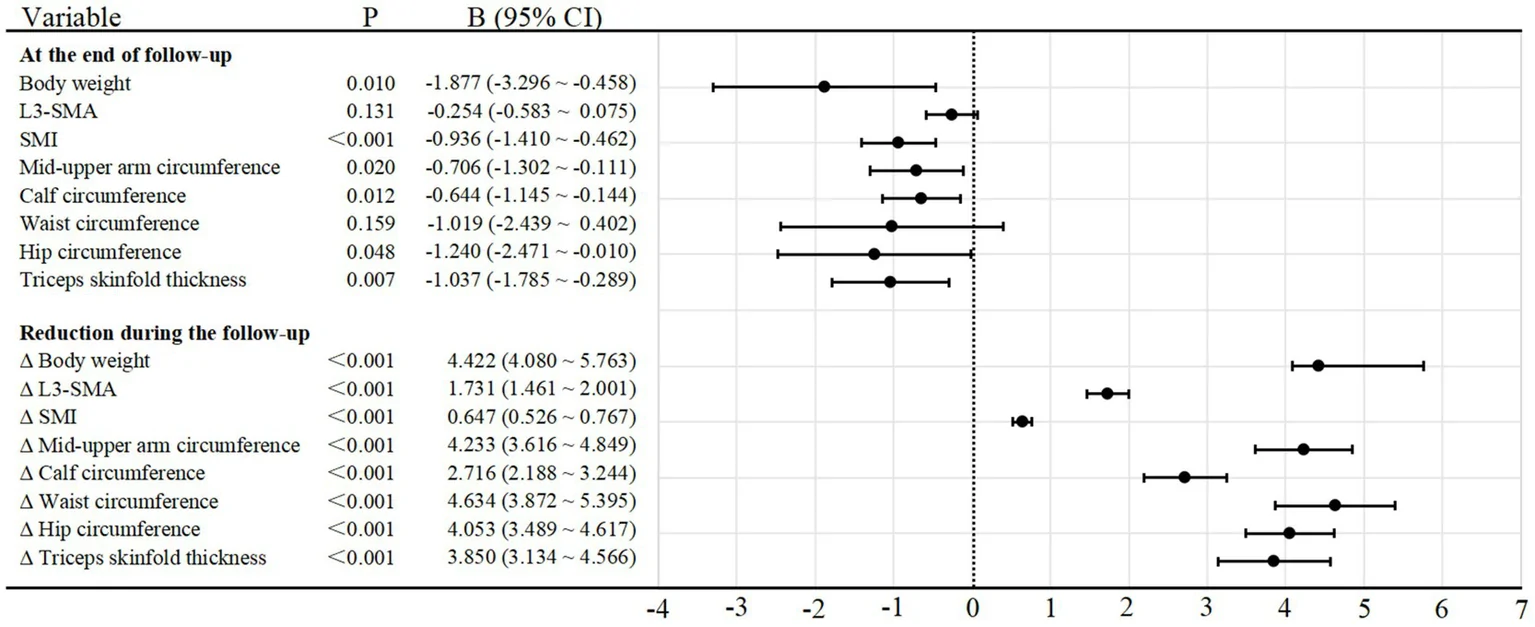
Association of postoperative DBFR with patients’ body weight, muscle mass, and anthropometric indicators during the follow-up. DBFR, delayed bowel function recovery; L3-SMA, skeletal muscle cross-sectional area at L3; SMI, skeletal muscle index. Association of postoperative delayed bowel function recovery with patients’ body weight, muscle mass, and nutrition-related indicators during the follow-up was determined using multivariate linear regression. A nominal association was defined as *p* < 0.05, and the corrected threshold for statistical significance was set at *p* < 0.003 (0.05/16).

Multivariate linear regression was adopted to explore associations of postoperative DBFR with body weight, muscle mass and nutritional indicators (including follow-up absolute values and their changes from baseline to endpoint). Postoperative DBFR was entered as a binary independent variable, whereas body composition and nutritional parameters were treated as continuous dependent variables. Given 16 separate regression analyses (Figure 1), Bonferroni correction was used for *p*-value adjustment. Raw *P* < 0.05 indicated nominal correlation, and the corrected significance cutoff was set at *P* < 0.003 (0.05/16).

Candidate covariates included baseline demographics (Table 1), preoperative serological markers (Supplementary Table 1), baseline body weight, muscle mass and anthropometric measurements (Table 2), tumor features and treatment protocols (Table 3), together with dietary intake and nutritional support during follow-up (Table 4). Variables showing intergroup differences at *P* < 0.10 were preliminarily screened. Among these screened variables, only covariates with proven clinical relevance to weight loss, muscle depletion, nutritional status or cancer cachexia were incorporated into the final regression model. Variables lacking clear clinical relevance were excluded to prevent redundant covariates and model overfitting. Variance inflation factor (VIF) was calculated to assess multicollinearity, with VIF < 10 indicating the absence of serious multicollinearity.

**Table 1:** Baseline characteristics of patients in the two groups.

Variable	DBFR (*n* = 177)	Non-DBFR (*n* = 441)	*t/χ* ^2^	*P*
Demographic data				
Male (n)	106 (59.9)	248 (56.2)	0.688	0.407
Age (year)	61.8 ± 9.2	59.9 ± 10.6	2.151	0.032
Body mass index (kg/m^2^)	22.4 ± 3.1	22.8 ± 3.5	1.562	0.119
Personal history				
Smoking (n)	46 (26.0)	95 (21.5)	1.418	0.234
Drinking (n)	41 (23.2)	83 (18.8)	1.485	0.223
Low-fiber diet (n)	74 (41.8)	146 (33.1)	4.171	0.041
High red meat intake (n)	58 (32.8)	129 (29.3)	0.740	0.390
Sedentary lifestyle (n)	63 (35.6)	144 (32.7)	0.490	0.484
Staying up late (n)	54 (30.5)	119 (27.0)	0.778	0.378
Disease history				
Hypertension (n)	81 (45.8)	162 (36.7)	4.315	0.038
Type 2 diabetes (n)	49 (27.7)	87 (19.7)	4.658	0.031
Coronary heart disease (n)	29 (16.4)	63 (14.3)	0.439	0.508
Stroke (n)	14 (7.9)	27 (6.1)	0.651	0.420
Intestinal polyps (n)	78 (44.1)	182 (41.3)	0.406	0.524
Inflammatory bowel disease (n)	5 (2.8)	11 (2.5)	0.055	0.815
Family history				
Intestinal polyps (n)	27 (15.3)	56 (12.7)	0.710	0.400
Colorectal cancer (n)	21 (11.9)	43 (9.8)	0.608	0.436

DBFR, Delayed bowel function recovery. Continuous variables are presented as mean ± standard deviation (SD), and the differences between groups are evaluated using the independent samples *t*-test. Categorical variables are expressed as frequency (constituent ratio), and the differences between groups are assessed by the chi-square (χ^2^) test. A *p* < 0.05 indicates statistical significance.

**Table 2:** Preoperative body weight, muscle mass, and anthropometric indicators of patients in the two groups.

Variable	DBFR (*n* = 177)	Non-DBFR (*n* = 441)	*t/χ* ^2^	*P*
Body weight/muscle mass				
Body weight (kg)	61.7 ± 8.2	62.8 ± 8.0	1.475	0.141
L3-SMA (cm^2^)	106.9 ± 19.0	108.2 ± 18.3	0.774	0.439
Body height (cm)	166.1 ± 10.7	166.5 ± 10.3	0.416	0.678
Skeletal muscle index (cm^2^/m^2^)	38.4 ± 2.5	38.7 ± 2.5	1.460	0.145
Anthropometric indicator				
Mid-upper arm circumference (cm)	24.6 ± 3.0	24.9 ± 3.6	0.960	0.337
Calf circumference (cm)	32.7 ± 2.6	33.1 ± 2.9	1.465	0.144
Waist circumference (cm)	79.1 ± 8.1	79.7 ± 8.1	0.753	0.452
Hip circumference (cm)	88.7 ± 6.7	89.5 ± 7.1	1.328	0.185
Triceps skinfold thickness (mm)	14.8 ± 4.0	15.4 ± 4.3	1.618	0.106

DBFR, Delayed bowel function recovery; L3-SMA, Skeletal muscle cross-sectional area at L3. Continuous variables are presented as mean ± standard deviation (SD), and the differences between groups are evaluated using the independent samples *t*-test. Categorical variables are expressed as frequency (constituent ratio), and the differences between groups are assessed by the chi-square (χ^2^) test. A *p* < 0.05 indicates statistical significance.

**Table 3:** Tumor characteristics and treatment measures of patients in the two groups.

Variable	DBFR (*n* = 177)	Non-DBFR (*n* = 441)	*t/χ* ^2^	*P*
Tumor location (n)				
Colon	98 (55.4)	259 (58.7)	0.585	0.444
Rectum	79 (44.6)	182 (41.3)		
Tumor diameter (n)				
≤ 5 cm	101 (57.1)	293 (66.4)	4.807	0.028
>5 cm	76 (42.9)	148 (33.6)		
Pathological type (n)				
Adenocarcinoma	164 (92.7)	407 (92.3)	0.024	0.877
Non-adenocarcinoma	13 (7.3)	34 (7.7)		
Differentiation degree (n)				
Moderate to high	121 (68.4)	336 (76.2)	4.018	0.045
Low to undifferentiation	56 (31.6)	105 (23.8)		
TNM stage (n)				
Stage I-II	93 (52.5)	286 (64.9)	8.070	0.004
Stage III	84 (47.5)	155 (35.1)		
Vascular invasion (n)	58 (32.8)	110 (24.9)	3.907	0.048
Perineural invasion (n)	51 (28.8)	96 (21.8)	3.458	0.063
Regional lymph node metastasis (n)	79 (44.6)	151 (34.2)	5.838	0.016
Carcinoembryonic antigen (ng/mL)	14.3 ± 7.8	12.8 ± 6.8	2.230	0.026
Surgical approach (n)				
Minimally invasive surgery	114 (64.4)	315 (71.4)	2.933	0.087
Open surgery	63 (35.6)	126 (28.6)		
Surgical resection extent (n)				
Right-sided colectomy	41 (23.2)	118 (26.8)	0.854	0.356
Left-sided colectomy	38 (21.5)	105 (23.8)	0.389	0.533
Anterior rectal resection	67 (37.9)	164 (37.2)	0.024	0.877
Abdominoperineal resection	22 (12.4)	42 (9.5)	1.149	0.284
Others	9 (5.1)	12 (2.7)	2.150	0.143
Surgical duration (n)				
≤180 min	108 (61.0)	290 (65.8)	1.239	0.266
>180 min	69 (39.0)	151 (34.2)		
ERAS compliance (n)				
Compliant	102 (57.6)	271 (61.5)	0.772	0.380
Non-compliant	75 (42.4)	170 (38.5)		
Perioperative opioid use (n)	96 (54.2)	223 (50.6)	0.681	0.409
Postoperative complication grading (n)				
Clavien-Dindo I-II	169 (95.5)	430 (97.5)	1.739	0.187
Clavien-Dindo III-V	8 (4.5)	11 (2.5)		
Postoperative complications (n)				
Incisional infection	17 (9.6)	36 (8.2)	0.335	0.563
Pulmonary infection	14 (7.9)	29 (6.6)	0.347	0.556
Intra-abdominal infection	9 (5.1)	16 (3.6)	0.690	0.406
Anastomotic leak	5 (2.8)	7 (1.6)	1.016	0.313
Postoperative chemotherapy (n)	104 (58.8)	224 (50.8)	3.216	0.073
Timing of chemotherapy (n)				
within 4–6 weeks	55 (52.9)	128 (57.1)	0.522	0.470
>6 weeks	49 (47.1)	96 (42.9)		
Intensity of chemotherapy (n)				
Standard dosage	51 (49.0)	120 (53.6)	0.585	0.444
Reduced dosage	53 (51.0)	104 (46.4)		
Postoperative radiotherapy (n)	25 (14.1)	52 (11.8)	0.630	0.427
Postoperative targeted therapy (n)	12 (6.8)	31 (7.1)	0.012	0.912

DBFR, Delayed bowel function recovery; TNM stage, Tumor, node, metastasis staging system; ERAS, Enhanced recovery after surgery. Continuous variables are presented as mean ± standard deviation (SD), and the differences between groups are evaluated using the independent samples *t*-test. Categorical variables are expressed as frequency (constituent ratio), and the differences between groups are assessed by the chi-square (χ^2^) test. A *P* < 0.05 indicates statistical significance.

**Table 4:** Dietary intake and nutritional support regimens of patients during the follow-up in the two groups.

Variable	DBFR (*n* = 177)	Non-DBFR (*n* = 441)	*t/χ* ^2^	*P*
Dietary fiber intake (g/d)	16.2 ± 4.2	16.7 ± 4.3	1.470	0.142
Protein intake (g/kg body weight/d)	1.0 ± 0.3	1.1 ± 0.3	2.546	0.011
Energy intake (kcal/kg body weight/d)	28.1 ± 3.6	28.9 ± 4.5	2.215	0.027
Protein supplement use (n)				
Present	38 (21.5)	105 (23.8)	0.389	0.533
Absent	139 (78.5)	336 (76.2)		
Vitamin/mineral supplement (n)				
Present	51 (28.8)	149 (33.8)	1.427	0.232
Absent	126 (71.2)	292 (66.2)		

DBFR, Delayed bowel function recovery. Continuous variables are presented as mean ± standard deviation (SD), and the differences between groups are evaluated using the independent samples *t*-test. Categorical variables are expressed as frequency (constituent ratio), and the differences between groups are assessed by the chi-square (χ^2^) test. A *P* < 0.05 indicates statistical significance.

Subgroup multivariate linear regression was further performed stratified by colon cancer versus rectal cancer, adopting identical covariates as the primary analysis. *P* < 0.05 denoted statistical significance.

All statistical computations were completed with SPSS 29.0.

## 3 Results

### 3.1 Patients

A total of 637 preoperative CRC candidates were initially screened. Nineteen patients were lost to follow-up owing to withdrawal, new-onset severe comorbidities or death, leaving a final analytic cohort of 618 patients (97.0%). Participants were categorized into DBFR group (*n* = 177) and non-DBFR group (*n* = 441) according to postoperative DBFR status.

### 3.2 Baseline characteristics

As shown in Table 1, patients in the DBFR group were older (*p* = 0.032), had a higher prevalence of low-fiber dietary habits (*p* = 0.041), hypertension (*p* = 0.038) and type 2 diabetes (*p* = 0.031) relative to the non-DBFR group.

### 3.3 Preoperative serological indicators

As shown in Supplementary Table 1, the DBFR group presented lower serum albumin (*p* = 0.013) but higher fasting blood glucose and C-reactive protein (*p* = 0.016, *p* = 0.027, respectively) versus the non-DBFR group.

### 3.4 Preoperative body weight, muscle mass, and anthropometric indicators

As shown in Table 2, no between-group differences were detected in preoperative body weight, skeletal muscle mass or anthropometric measurements (all *p* > 0.05).

### 3.5 Tumor characteristics and treatment measures

As shown in Table 3, the DBFR group had higher rates of tumor diameter >5 cm, poor to undifferentiated histology, stage III disease, vascular invasion and lymph node metastasis, alongside elevated preoperative carcinoembryonic antigen levels (*p* = 0.028, *p* = 0.045, *p* = 0.004, *p* = 0.048, *p* = 0.016, *p* = 0.026, respectively).

### 3.6 Dietary intake and nutritional support regimens during the follow-up

As shown in Table 4, daily protein and energy intake were significantly lower in the DBFR group (*p* = 0.011, *p* = 0.027).

### 3.7 Body weight, muscle mass, and anthropometric indicators during the follow-up

As shown in Table 5, at the 6-month follow-up, the DBFR group exhibited lower body weight (*p* = 0.009), reduced SMI (*p* < 0.001), shorter mid-upper arm, calf and hip circumferences (*p* = 0.018, *p* = 0.011, *p* = 0.049), and thinner triceps skinfold thickness (*p* = 0.009) compared with controls.

**Table 5:** Body weight, muscle mass, and anthropometric indicators of patients during the follow-up in the two groups.

Variable	DBFR (*n* = 177)	Non-DBFR (*n* = 441)	*t/χ* ^2^	*P*
At the end of follow-up				
Body weight (kg)	59.0 ± 8.3	60.9 ± 8.0	2.636	0.009
L3-SMA (cm^2^)	101.8 ± 19.2	104.8 ± 18.5	1.809	0.071
SMI (cm^2^/m^2^)	36.5 ± 2.8	37.4 ± 2.7	3.985	<0.001
Mid-upper arm circumference (cm)	23.3 ± 3.0	24.0 ± 3.5	2.365	0.018
Calf circumference (cm)	31.8 ± 2.7	32.4 ± 2.9	2.535	0.011
Waist circumference (cm)	77.5 ± 8.2	78.5 ± 8.1	1.390	0.165
Hip circumference (cm)	87.5 ± 6.8	88.7 ± 7.2	1.971	0.049
Triceps skinfold thickness (mm)	13.4 ± 4.1	14.4 ± 4.3	2.624	0.009
Reduction during the follow-up				
Δ Body weight (kg)	2.7 ± 1.0	1.9 ± 0.6	12.331	<0.001
Δ L3-SMA (cm^2^)	5.1 ± 2.1	3.4 ± 1.3	12.595	<0.001
Δ SMI (cm^2^/m^2^)	1.9 ± 0.8	1.2 ± 0.6	10.551	<0.001
Δ Mid-upper arm circumference (cm)	1.3 ± 0.5	0.9 ± 0.3	13.478	<0.001
Δ Calf circumference (cm)	1.0 ± 0.4	0.7 ± 0.2	10.105	<0.001
Δ Waist circumference (cm)	1.7 ± 0.6	1.2 ± 0.3	11.951	<0.001
Δ Hip circumference (cm)	1.2 ± 0.5	0.8 ± 0.2	14.110	<0.001
Δ Triceps skinfold thickness (mm)	1.4 ± 0.6	1.0 ± 0.3	10.566	<0.001

DBFR, Delayed bowel function recovery; L3-SMA, Skeletal muscle cross-sectional area at L3; SMI, Skeletal muscle index. Continuous variables are presented as mean ± standard deviation (SD), and the differences between groups are evaluated using the independent samples *t*-test. Categorical variables are expressed as frequency (constituent ratio), and the differences between groups are assessed by the chi-square (χ^2^) test. A *P* < 0.05 indicates statistical significance.

Notably, the DBFR group experienced significantly larger declines from baseline to follow-up in body weight, L3-SMA, SMI, mid-upper arm circumference, calf circumference, waist circumference, hip circumference and triceps skinfold thickness (all *p* < 0.001).

### 3.8 Association of postoperative DBFR with patients’ body weight, muscle mass, and anthropometric indicators during the follow-up

Based on the prespecified covariate screening criteria, 20 variables with intergroup *p* < 0.10 were preliminarily included. Fourteen clinically relevant covariates closely linked to weight loss, muscle wasting, nutritional status and cancer cachexia were retained for final modeling: age, low-fiber diet, hypertension, type 2 diabetes, albumin, fasting blood glucose, C-reactive protein, tumor diameter, tumor differentiation, TNM stage, vascular invasion, lymph node metastasis, carcinoembryonic antigen and postoperative chemotherapy. VIF values for all included variables were <10, indicating no substantial multicollinearity. All 14 covariates were entered into multivariate linear regression models.

As shown in Figure 1, multivariate linear regression revealed that postoperative DBFR was nominally associated with reduced follow-up body weight (*B* = −1.877, 95%CI = −3.296 ~ −0.458, *p* = 0.010) and significantly associated with lower SMI (B = −0.936, 95%CI = −1.410 ~ −0.462, *p* < 0.001). DBFR also showed nominal correlations with shorter mid-upper arm circumference (*p* = 0.020), calf circumference (*p* = 0.012) and hip circumference (*p* = 0.048), and a significant correlation with thinner triceps skinfold thickness (*p* = 0.007).

Furthermore, DBFR was significantly linked to greater reductions in body weight and SMI from baseline to follow-up (*B* = 4.422, 95%CI = 4.080 ~ 5.763, *P <* 0.001; *B* = 0.647, 95%CI = 0.526 ~ 0.767, *P <* 0.001), as well as significant declines in mid-upper arm circumference, calf circumference, waist circumference, hip circumference and triceps skinfold thickness (all *p* < 0.001).

### 3.9 Subgroup analysis by colon versus rectal cancer: association of DBFR with weight and muscle mass

As shown in Supplementary Table 2, subgroup analyses stratified by colon and rectal cancer demonstrated consistent associations between DBFR and changes in body weight and SMI relative to the overall cohort results.

## 4 Discussion

DBFR is a common postoperative complication after CRC resection (20). Postoperative weight and muscle loss are well-established prognostic indicators in CRC patients (21). This is the first study to explore the independent association between postoperative DBFR and subsequent weight and muscle loss, with potential scientific and clinical implications.

A core finding showed that postoperative DBFR was independently associated with lower body weight at the 6-month follow-up, which was consistently supported by multiple body composition measurements. Specifically, patients with DBFR presented significantly reduced SMI. Meanwhile, anthropometric markers reflecting nutritional reserve, including mid-upper arm circumference, calf circumference and triceps skinfold thickness, were nominally or significantly lower in the DBFR group. Collectively, consistent results across body weight, skeletal muscle mass and anthropometric parameters indicate that postoperative DBFR is correlated with impaired nutritional status and skeletal muscle wasting at 6 months after surgery.

Furthermore, our data demonstrated that DBFR was related to greater declines in body weight, SMI and anthropometric indices from baseline to follow-up; the low corresponding *p* values confirmed the high statistical reliability of the results. Compared with single time-point absolute values, baseline-adjusted changes help minimize intergroup baseline confounding and better reflect the independent association between DBFR and progressive nutritional and skeletal muscle depletion. Clinically, these findings suggest DBFR is not merely an early transient intestinal disorder, but correlates with sustained 6-month muscle loss and nutritional deterioration. If validated in future large-scale multicenter trials, DBFR may serve as a practical clinical marker to identify patients at high risk of sarcopenia and malnutrition and assist in designing personalized nutritional and muscle-protective interventions.

Univariate analyses revealed that advanced age, comorbid hypertension and type 2 diabetes, preoperative hypoalbuminemia, elevated CRP and advanced tumor stage were correlated with postoperative DBFR. Pathophysiologically, advanced age and chronic diseases may weaken intestinal peristaltic reserve; preoperative hypoalbuminemia may reflect insufficient nutritional storage; elevated CRP may mirror exaggerated perioperative inflammation; advanced tumor stage is often accompanied by heightened systemic inflammatory burdens and catabolism, all of which may collectively contribute to delayed bowel function recovery (22–25). Notably, these clinical characteristics are also linked to aggravated postoperative weight and muscle loss (26–29), acting as important confounders when exploring the association between DBFR and body composition outcomes. Our identified associated factors also provide clues for future research exploring the potential linkage between DBFR and nutritional as well as muscle metabolic disorders.

We constructed multivariate linear regression models incorporating 14 clinically relevant confounders covering baseline demographics, preoperative serology, tumor features and treatment modalities. Covariates were selected via *p* < 0.10 univariate screening combined with established clinical evidence rather than automated stepwise selection; VIF testing was performed to exclude substantial multicollinearity. This rigorous confounding adjustment reliably confirmed the independent association between DBFR and adverse body composition outcomes.

Strict predefined inclusion and exclusion criteria restricted enrollment to stage I–III patients eligible for curative resection; individuals with severe comorbidities, cognitive impairment, incomplete follow-up or missing core data were excluded. Rigorous cohort selection guaranteed sample homogeneity and minimized residual confounding bias.

CT-derived SMI quantified via standardized ImageJ segmentation represents a practical muscle-assessment approach: routine postoperative surveillance CT enables convenient data acquisition, and SMI reflects skeletal muscle reserve and nutritional status more precisely than crude body weight or subjective physical evaluation, allowing detection of subtle postoperative muscle loss.

Our 72-h flatus/defecation-based DBFR definition carries inherent classification bias; the broad diagnostic threshold may include mild gastrointestinal dysfunction cases that do not meet strict criteria such as GI-2. The conservative nature of this definition may tend to underestimate the magnitude of the actual association between DBFR and 6-month nutritional outcomes. Despite this potential attenuation of the association, significant associations remained detectable, further confirming the robustness of our findings.

Although DBFR presents as an early transient postoperative intestinal dysfunction, it shows an association with sustained 6-month weight and muscle loss in CRC patients, with two plausible mechanistic links: (1) early metabolic dysregulation accompanied by delayed gut function may impair intestinal digestion and nutrient utilization, potentially triggering adaptive skeletal muscle catabolism to maintain basal metabolism and gradual muscle depletion regardless of postoperative nutritional support. (2) Transient intestinal barrier alteration related to DBFR may be accompanied by low-grade persistent inflammation. Even after the recovery of intestinal motility, sustained activation of inflammatory catabolic pathways may inhibit muscle synthesis, possibly extending the unfavorable profile of early gut dysfunction to 6 months postoperatively and coinciding with sustained weight and muscle loss. Further basic studies are required to verify these speculative mechanistic correlations.

Several limitations of this study should be acknowledged. First, oncologic survival endpoints were not analyzed. However, prior literature has consistently verified sarcopenia and weight loss as poor prognostic markers for CRC (30). Rather than repeating these established associations, our study focused on exploring the linkage within the pathway of DBFR and altered body composition, which may indirectly imply a plausible sequential prognostic correlation: postoperative DBFR → altered body composition → unfavorable prognosis. Further long-term follow-up studies are warranted to verify this sequential prognostic relevance.

Other limitations include: single-center enrollment that may limit external generalizability due to region-specific patient characteristics and institutional perioperative protocols (31); reliance solely on CT-derived muscle mass without functional testing, whereas sarcopenia involves both quantitative muscle mass and physical function; a six-month follow-up period is relatively short to reflect long-term progressive body composition changes; residual unmeasured confounders such as home rehabilitation compliance and gut microbiota composition shifts may potentially interfere with the observed associations (32); and the absence of translational laboratory experiments to clarify the underlying biological correlations. These limitations are largely attributable to practical research constraints. Future multicenter prospective studies with comprehensive covariate collection and mechanistic animal and cell experiments will help refine and extend our observations.

In conclusion, postoperative DBFR is independently associated with medium-term weight and skeletal muscle loss at 6 months after CRC resection. Upon further external validation, DBFR may serve as an early predictive marker for identifying patients at high risk of subsequent nutritional deterioration and muscle wasting, facilitating targeted postoperative surveillance and personalized early supportive interventions to improve patients’ nutritional prognosis.

## Data Availability

The raw data supporting the conclusions of this article will be made available by the authors, without undue reservation.
