# Supplementary material for: Association of postoperative delayed bowel function recovery with weight and muscle loss at 6 months in colorectal cancer patients
Source: Front Nutr. 2026 Aug 7;13:1827831. doi: 10.3389/fnut.2026.1827831 (PMC13493217; doi:10.3389/fnut.2026.1827831)
Supplement: Supplementary file 1 [file Supplementary_file__1.docx]

**Supplemental Figure 1 Flowchart of the present study**

Note: CRC = Colorectal cancer, DBFR = Delayed bowel function recovery.

**Supplemental Table 1 Preoperative serological indicators of patients in the two groups**

| **Variable** | **DBFR**  **(n = 177)** | **Non-DBFR**  **(n = 441)** | ***t / χ²*** | ***P*** |
| --- | --- | --- | --- | --- |
| **Complete blood count** |  |  |  |  |
| Hemoglobin (g/L) | 120.7 ± 16.4 | 122.6 ± 16.0 | 1.310 | 0.191 |
| White blood cell count (×10^9^/L) | 7.7 ± 2.5 | 7.9 ± 2.2 | 1.326 | 0.185 |
| **Coagulation function** |  |  |  |  |
| Prothrombin time (s) | 12.5 ± 1.3 | 12.4 ± 1.0 | 1.461 | 0.145 |
| Fibrinogen (g/L) | 3.8 ± 1.1 | 3.6 ± 0.9 | 1.667 | 0.096 |
| **Liver and kidney function** |  |  |  |  |
| Albumin (g/L) | 39.4 ± 4.4 | 40.3 ±4.2 | 2.487 | 0.013 |
| Total bilirubin (μmol/L) | 12.5 ± 5.3 | 11.7 ± 4.7 | 1.708 | 0.088 |
| Creatinine (μmol/L) | 74.2 ± 19.9 | 72.9 ± 17.3 | 0.821 | 0.412 |
| **Blood lipids and glucose** |  |  |  |  |
| Total cholesterol (mmol/L) | 4.7 ± 1.0 | 4.6 ± 0.9 | 1.829 | 0.068 |
| Triglyceride (mmol/L) | 1.7 ± 0.7 | 1.6 ± 0.7 | 1.412 | 0.159 |
| Fasting blood glucose (mmol/L) | 6.5 ± 1.6 | 6.2 ± 1.2 | 2.420 | 0.016 |
| **Electrolytes** |  |  |  |  |
| Potassium (mmol/L) | 4.2 ±0.5 | 4.3 ± 0.5 | 1.810 | 0.071 |
| Sodium (mmol/L) | 140.4 ± 3.5 | 140.0 ± 3.0 | 1.230 | 0.219 |
| **Inflammation** |  |  |  |  |
| C-reactive protein (mg/L) | 17.7 ± 9.1 | 16.1 ± 7.6 | 2.220 | 0.027 |

Note: DBFR = Delayed bowel function recovery. Continuous variables are presented as mean±standard deviation (SD), and the differences between groups are evaluated using the independent samples t-test. Categorical variables are expressed as frequency (constituent ratio), and the differences between groups are assessed by the chi-square (χ²) test. A P-value＜0.05 indicates statistical significance.

**Supplemental Table 2 Subgroup analysis by colon versus rectal cancer: association of DBFR with weight and muscle mass**

| **Variable** | **Multivariate linear regression** | | |
| --- | --- | --- | --- |
|  | ***P*** | ***B*** | ***95% CI*** |
| **Colon cancer** |  |  |  |
| Body weight at the end of follow-up | 0.022 | -1.742 | -3.215 ~ -0.269 |
| SMI at the end of follow-up | ＜0.001 | -0.885 | -1.342 ~ -0.428 |
| Δ Body weight during the follow-up | ＜0.001 | 4.216 | 3.892 ~ 5.540 |
| Δ SMI during the follow-up | ＜0.001 | 0.612 | 0.495 ~ 0.729 |
| **Rectal cancer** |  |  |  |
| Body weight at the end of follow-up | 0.031 | -2.015 | -3.583 ~ -0.447 |
| SMI at the end of follow-up | ＜0.001 | -0.984 | -1.456 ~ -0.512 |
| Δ Body weight during the follow-up | ＜0.001 | 4.685 | 4.293 ~ 6.077 |
| Δ SMI during the follow-up | ＜0.001 | 0.683 | 0.558 ~ 0.808 |

Note: DBFR = Delayed bowel function recovery, SMI = Skeletal muscle index. Association of postoperative delayed bowel function recovery with patients' body weight and muscle mass during the follow-up was determined using multivariate linear regression. A P-value＜0.05 indicates statistical significance.
